# A method for calculating vector forces at human-mattress interface during sleeping positions utilizing image registration

**DOI:** 10.1038/s41598-024-66035-8

**Published:** 2024-07-02

**Authors:** Ying Gao, Jing Zhang, Chengzhao Zou, Liwen Bi, Chengzhen Huang, Jiachen Nie, Yongli Yan, Xinli Yu, Fujun Zhang, Fanglai Yao, Li Ding

**Affiliations:** 1 Beijing Advanced Innovation Center for Biomedical Engineering, Key Laboratory for Biomechanics and Mechanobiology of Ministry of Education, School of Biological Science and Medical Engineering, Beihang University, Beijing, 100191 China; 2 De Rucci Healthy Sleep Co., Ltd, Dongguan, 523960 Guangdong China

**Keywords:** Vector forces, Sleeping position, Image registration, Musculoskeletal simulations, Biomedical engineering, Quality of life

## Abstract

The vector forces at the human-mattress interface are not only crucial for understanding the distribution of vertical and shear forces exerted on the human body during sleep but also serves as a significant input for biomechanical models of sleeping positions, whose accuracy determines the credibility of predicting musculoskeletal system loads. In this study, we introduce a novel method for calculating the interface vector forces. By recording indentations after supine and lateral positions using a vacuum mattress and 3D scanner, we utilize image registration techniques to align body pressure distribution with the mattress deformation scanning images, thereby calculating the vector force values for each unit area (36.25 mm × 36.25 mm). This method was validated through five participants attendance from two perspectives, revealing that (1) the mean summation of the vertical force components is 98.67% ± 7.21% body weight, exhibiting good consistency, and mean ratio of horizontal component force to body weight is 2.18% ± 1.77%. (2) the predicted muscle activity using the vector forces as input to the sleep position model aligns with the measured muscle activity (%MVC), with correlation coefficient over 0.7. The proposed method contributes to the vector force distribution understanding and the analysis of musculoskeletal loads during sleep, providing valuable insights for mattress design and evaluation.

## Introduction

Insufficient sleep has been linked to a heightened risk of cardiovascular disease, psychological depression, and musculoskeletal injuries^1–6^. A survey has revealed that an uncomfortable mattress can disrupt the distribution of spinal loads during sleep, accounting for 7% of sleep-related issues^7^. Poor mattress design may lead to concentrated pressure on specific body parts, such as the hips, shoulders, and back^8^, potentially affecting peripheral blood circulation and causing discomfort, including numbness and pain^9,10^. Therefore, it is imperative to consider the provision of adequate comfort and support during sleep when designing a high-quality mattress. This necessitates the harmonious spinal alignment^11,12^, appropriate joint position^13^, along with the promotion of muscle relaxation throughout the body^14^. Researchers have conducted extensive biomechanical research on sleeping positions^5,6^, such as conducting subjective surveys on preferred sleeping positions among people; using X-ray techniques, analyze the differences in spinal alignment between sleeping position and standing position^4,15^; evaluate the effect of different mattresses on sleep comfort using surface electromyography (sEMG) technology^16–20^. Research has shown that the differences in internal muscle and joint forces caused by distinct mattresses supports are one of the primary contributors affecting sleep comfort in the human body^21–24^.

The musculoskeletal modeling technology, especially the multi-rigid-body modelling technology (AnyBody, OpenSim, and LifeMod software, etc.), offer a comprehensive approach to analyzing musculoskeletal loads. These techniques provide a convenient tool for studying muscle and joint loads in the human body by establishing a human–environment mechanics simulation model^17^. While multi-rigid-body musculoskeletal modeling techniques have gained widespread use as a practical tool across diverse domains^25–27^, their reliability necessitates prior verification^28–30^. Given that the forces exerted at the human-mattress interface, as a boundary condition input for the sleeping posture model, are primary determinants of the loads on the human skeletal muscle system^14–17,21^, an efficient, noninvasive, and cost-effective acquisition of vector forces at the human-mattress interface is crucial. This is essential for enhancing the accuracy of inverse dynamic calculation results and expanding the application of modeling and simulation technology in mattress design optimization.

Understanding the interaction of forces between people and the beds they lie in can provide a deeper understanding of the load on their muscles, bones, and soft tissues^31^. Body pressure distribution testing is currently a common method for evaluating sleep mechanics between humans and mattresses^5,32^. Based on the consensus that force triggers skin deformation, and localized high pressure stimulates tactile and pain receptors, inducing discomfort and pain^33,34^, mattress design often aims to achieve a uniform distribution of contact pressure and minimize peak pressure^8,35–37^. However, traditional pressure mats can only measure scalar forces at the human-mattress interface, failing to directly capture the localized shear force^38^. The emergence of shear force during sleep often stems from varied indentation degrees resulting from body pressure distribution on the mattress and mattress surface deformation due to deep tissue adaptation to peak pressure points (e.g., heels, pelvis, and shoulders)^13^. Studies reveal that the combined influence of local skin shear force and normal force dictates the maximal stress and strain of internal tissues^38–40^.

Notably, there have been limited experimental methods that can simultaneously quantifying both normal and shear contact forces^41,42^. Goossens et al.^31^ developed a measurement device that records the vertical and parallel forces experienced by the human body when sitting on a bed with the back inclined at 45 degrees to the horizontal plane, across three independently adjustable body support surfaces. The measurements on a healthy population (77 ± 17 kg) showed an accuracy of 2% and the total shear force on the seat pan was 97 ± 17 N. Denninger et al.^28^ designed a mattress construction consisted of an array of eighteen rows and seven columns of foam cubes, which can customize the mattress and reduce torso shear force by balancing the weight of each body part with the support force provided by the corresponding row of the mattress. While this approach does not directly measure shear force values, it reduces shear force indirectly. Regarding shear force measurements between the body and seats, Bush and Hubbard^43^ and Beurier et al.^44^ developed experimental seats to measure both normal and shear forces. These seats can simulate a wide range of seat geometric configurations and measure contact forces, including both normal and shear components, which has significantly advanced the biomechanical analysis of sitting postures. Nevertheless, there is still limited research considering the shear force at the human-mattress interface.

This study introduces a method for calculating the vector force at the human-mattress interface using image registration technology based on body pressure distribution and scanned images of mattress deformation. This approach facilitates understanding the distribution of vector forces applied to the human body during sleep and can serve as an input for biomechanical models of sleeping positions, which helps to further analyze the internal musculoskeletal forces in the human body. The purpose of this paper is to describe the implementation details of the novel method and verify its effectiveness. We hypothesize that the method can be validated by the experiment with five subjects from two perspectives: (1) The sum of all vertical forces is calculated and compared to the subjects' weight, while the sum of all horizontal forces is calculated and compared to zero. (2) The vector forces calculated by this method are used as the boundary condition input for the sleeping model, and the correlation coefficient between predicted and measured muscle activity is not less than 0.7.

## Methods

### Subjects

Five healthy young individuals were recruited for this study, and their characteristics are detailed in Table 1^45^. Prior to the experiment, each participant read and provided written informed consent. The study was approved by the Ethics Committee of Beihang University and adhered to the principles outlined in the Helsinki Statement.

**Table 1:** Body characteristics of subjects.

Subject number	Gender	Height (cm)	Weight (kg)	BMI	Somatotype
1	Female	149	35	15.77	Underweight
2	Female	151	46	20.17	Normal
3	Male	186	63	18.21	Underweight
4	Male	182	69	20.83	Normal
5	Male	178	75	23.67	Normal

### Instrumentation

A vacuum positioning mattress (2,991,163/V, Stabilo Grande by AAT, Poland) was employed to preserve the soft tissue deformations of the human body during sleep. This mattress exhibits a unique characteristic: it begins in a highly pliable state before air is pumped into it, rendering it susceptible to deformation. However, by adjusting the air extraction, the mattress can be tailored to achieve the desired firmness, thereby becoming more resistant to further deformation. This attribute is instrumental in maintaining the contours of the human body's soft tissues.

A pressure mat (DeRUCCI MFD1-007/Smart Pressure Test Mat/Premium Edition, DeRUCCI Co. Ltd., China) with 1152 sensors distributed over an area of 1880*990 mm was utilized to record body pressure distribution data. The testing accuracy of this device is 1 mm Hg.

A handheld 3D scanner (Go! SCAN 50, CREAFORMTM, Canada) was used to capture indentations on the vacuum positioning mattress, providing detailed three-dimensional data of the surface contours.

A surface EMG recording System(Noraxon Corporation, TeleMyo^®^ 2400DTS, USA) was used for sEMG acquisition with a sampling frequency of 1500 Hz^46^.

### Experimental procedure

Prior to commencing the experiment, sEMG electrodes were placed according to the SENIAM standard^47^ on the erector spinae, multifidi, gluteus medius, tensor fasciae latae, and biceps femoris muscles, where the skin had been cleaned with alcohol and shaved. Adhesive bipolar surface electrodes (Noraxon USA Corporation, USA) were affixed above the midpoint of each analyzed muscle belly, ensuring their alignment was parallel to the presumed direction of the muscle fibers. Additionally, all electrodes and sensors were connected through connection wires and securely fastened using adhesive tape to minimize the occurrence of movement artifacts^30^. All examined muscles were evaluated bilaterally. The maximal voluntary contraction (MVC) of muscles were measured independently based on the SENIAM recommendations. The participants were required to exert their maximal effort of isometric strength for each of these muscles for approximately 5 s, and 3 measurements were made. A rest period of 5 min was given after each successful MVC measurement.

The surface of the vacuum positioning mattress was meticulously leveled to eliminate any potential artifacts in the pressure mapping. Subsequently, the mattress was firmed using an air pump to prevent undesired deformations during body positioning adjustments. A pressure mat was then positioned on the mattress to capture pressure distribution across various sleeping postures. The experimental protocol consisted of the following steps:

1. The subject was instructed to assume either a supine or lateral sleeping position on the pressure mat.
2. After confirming the posture and positioning, the vacuum mattress was inflated until the subject deemed it comfortably firm, comparable to their preferred mattress hardness.
3. Body pressure data from the pressure pad and sEMG readings from the six muscles were simultaneously recorded for a duration of 30 s.
4. With assistance from the experimenters, the subject arose, and the surface contours of the vacuum mattress were documented using a 3D scanner followed by full hardening through air extraction.

### Calculation of vector forces

In the data recorded by the pressure pad, a 24*48 matrix represents the arrangement position of the sensors, and store the pressure data. We utilize the Seaborn Python visualization library, which is built on top of matplotlib, to generate a heatmap from the pressure pad data matrix. To mitigate the impact of abrupt pressure changes, the middle 10 s of a 30-s pressure datasets were selected. Subsequently, the average pressure recorded by each sensor was computed. Utilizing collected data, including coordinates, normal vectors, point counts along each axis, and maximum/minimum values on each axis, the pressure mat data was expanded into a three-dimensional point cloud (pressure mat point cloud), matching the format of the vacuum mattress storage data. To facilitate registration, the pressure data which served as the axis data source needs to be normalized to scale the point cloud. The normalized pressure value is calculated using the formula:

1 $$F^{\prime} = {{ - F_{{x_{i} ,y_{i} }} } \mathord{\left/ {\vphantom {{ - F_{{x_{i} ,y_{i} }} } {F_{\max } }}} \right. \kern-0pt} {F_{\max } }}\left( {Z_{\max } - Z_{\min } } \right),$$

where $F^{\prime}$ represents the normalized pressure value, $F_{\max }$ is the maximum value in the initial pressure value, and $Z_{\max }$ and $Z_{\min }$ are the maximum and minimum values in the $z$-axis direction of the vacuum positioning mattress.

The 3D scanning image depicting the surface deformation of the vacuum mattress was saved in .ply point cloud format (vac-mat point cloud). The coordinate system's origin was set at the centroid of the mattress, with the x, y, and z axes aligning with the width, length, and height of the mattress, respectively.

**Figure 1:**
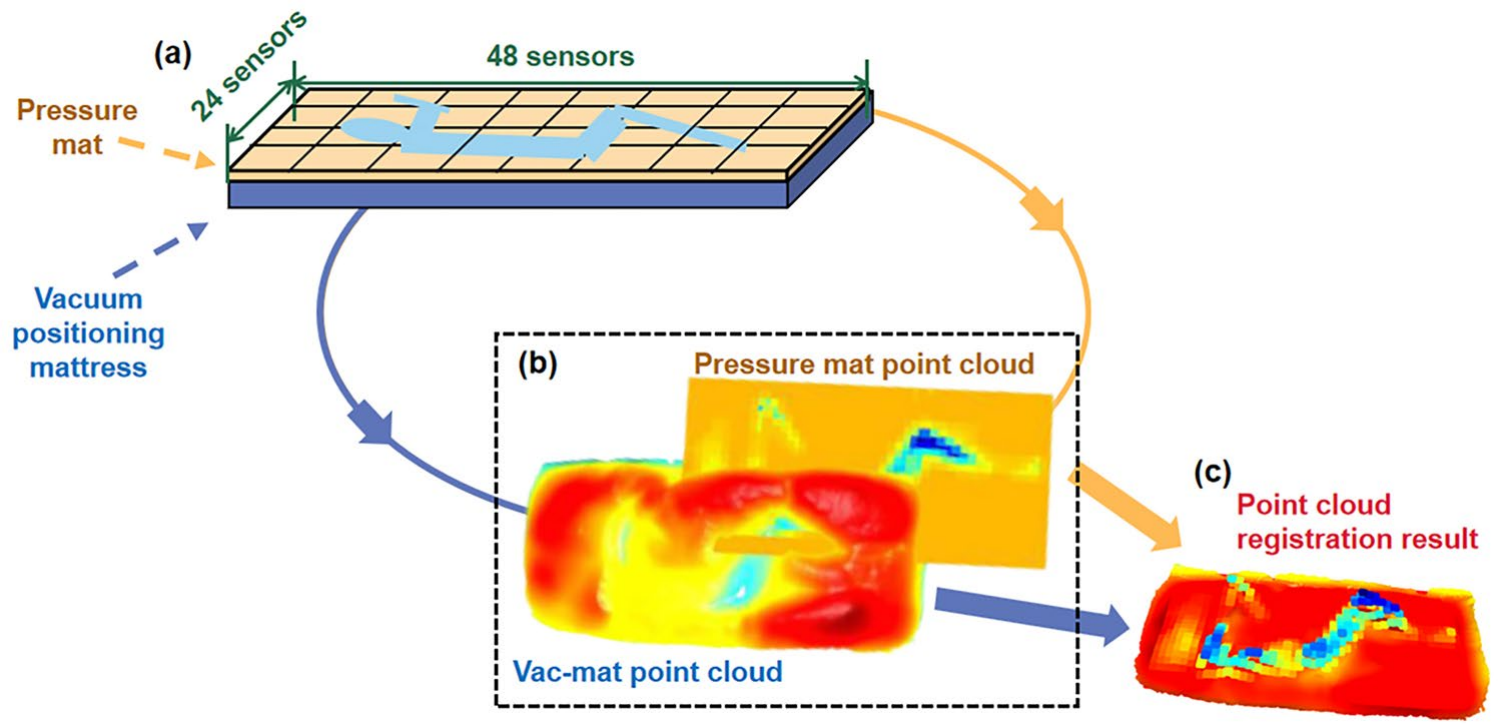
Registration process of pressure mat point cloud and vac-mat point cloud.

Considering the pressure mat point cloud as the current point cloud and the vac-mat point cloud as the target, the pressure mat point cloud was translated, rotated, and further adjusted based on the visualization image to achieve point cloud registration. Figure 1 illustrates the point cloud pairing process. The point clouds described in (b), "Pressure mat point cloud" and "Vac-mat point cloud" are not aligned in the same coordinate system and cannot be used for vector force calculations. The goal of point cloud registration is to align the coordinate systems, determining the coordinate transformation relationship and thus the positional relationship between the pressure data and the mattress surface deformation data. Open3D utilizes the Iterate Closest Point (ICP) algorithm for point cloud registration. Considering the pressure mat point cloud as the current point cloud and the vac-mat point cloud as the target, the pressure mat point cloud was translated, rotated, and further adjusted based on the visualization image to achieve point cloud registration. After registration, the visualization function in Open3D allows for viewing the results, facilitating a better understanding of the alignment effect of the point cloud data. Figure (c) illustrates the result after point cloud registration.

As the initial pressure $F$ measured by the pressure mat sensors are actually scalar forces perpendicular to the plane of the sensor. Each sensor of the pressure mat corresponds to a small cell size of 36.25 mm × 36.25 mm. For each cell of the pressure mat, the number N of points in the vac-mat point cloud can be determined, along with the corresponding normal vector $\left( {x_{i} ,y_{i} ,z_{i} } \right)$ for each point. The average of these normal vectors is taken as the representative normal vector for each cell. These normal vectors $\overrightarrow{\text{n}}$ are then converted to unit normal vectors $\vec{n}_{s}$.

2 $${{x_{n} = \sum\limits_{x}^{N} {x_{i} } } \mathord{\left/ {\vphantom {{x_{n} = \sum\limits_{x}^{N} {x_{i} } } N}} \right. \kern-0pt} N}\,\,{{y_{n} = \sum\limits_{x}^{N} {y_{i} } } \mathord{\left/ {\vphantom {{y_{n} = \sum\limits_{x}^{N} {y_{i} } } N}} \right. \kern-0pt} N}\,\,{{z_{n} = \sum\limits_{x}^{N} {z_{i} } } \mathord{\left/ {\vphantom {{z_{n} = \sum\limits_{x}^{N} {z_{i} } } N}} \right. \kern-0pt} N},$$

3 $$\vec{n} = \left( {x_{n} ,y_{n} ,z_{n} } \right),$$

4 $$\vec{n}_{s} = {{\vec{n}} \mathord{\left/ {\vphantom {{\vec{n}} {\left| {\vec{n}} \right|}}} \right. \kern-0pt} {\left| {\vec{n}} \right|}}.$$

By obtaining the initial pressure $F$ and coordinates $\vec{n}_{s}$ for each cell, the vector force $\mathop{F}\limits^{\rightharpoonup} _{s}$ corresponding to each cell can be calculated.

5 $$\vec{F}_{S} = F\vec{n}_{s}$$

### Validation

#### Calculation of the sum of vertical and horizontal force components

The gravitational force acting on the human body during sleep should match closely with the sum of the vertical components of the vector forces. The gravitational force *G* can be calculated from the body weight of each subject, and the total vertical force *F*_*z*_ can be obtained using the equation provided below.

6 $${F}_{Z} = \sum_{i}{f}_{{z}_{i}}$$

**Figure 2:**
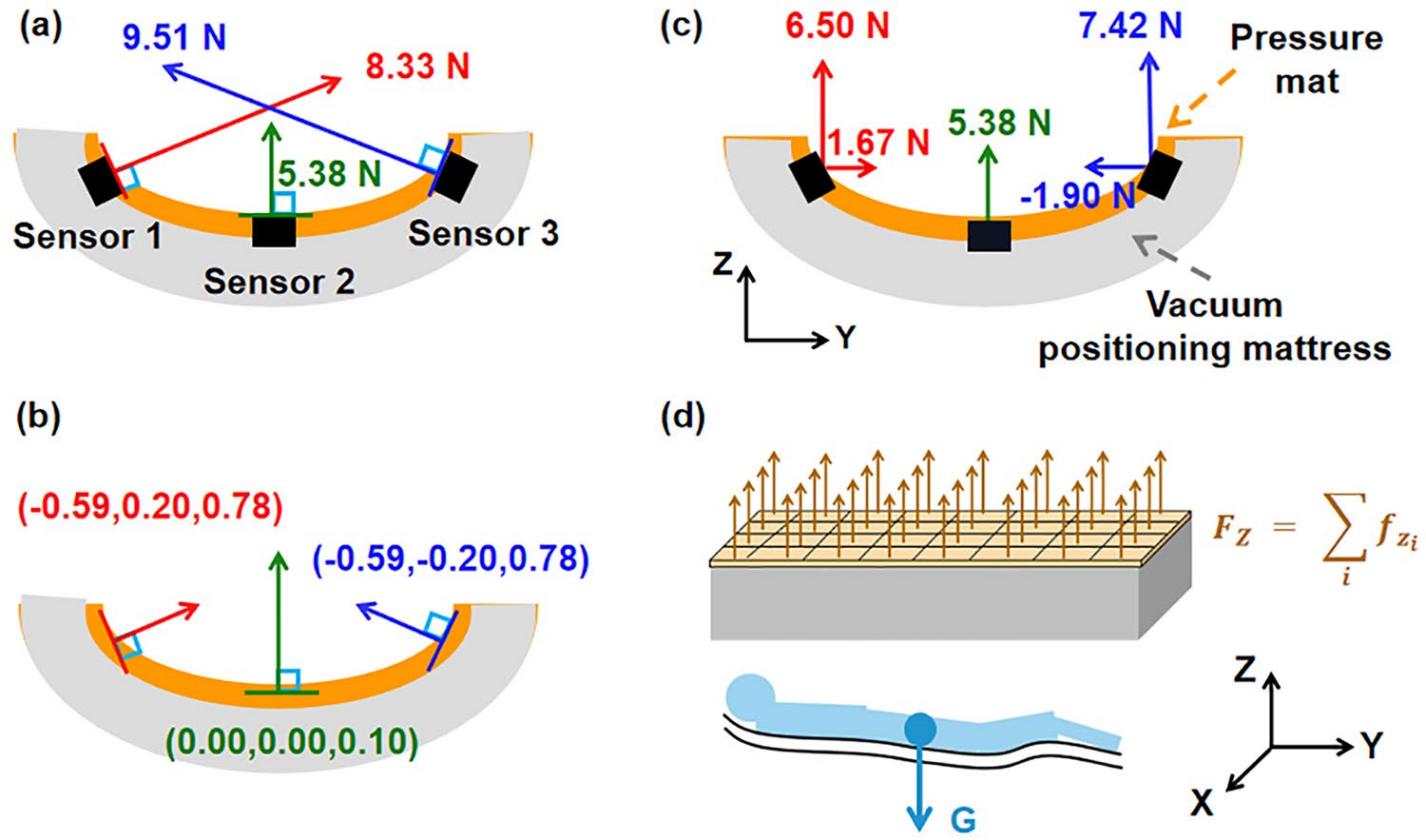
Calculation process of the sum of vertical force components.

The calculation process of the sum of vertical force components is illustrated in Fig. 2, divided into four steps: (a)The scalar force measured by the sensor displayed on the z-x plane; (b)The average normal vector at the position where the sensor is located; (c)The vertical components of the vector forces. The vector forces calculated from the scalar forces and the average normal vector shown in (a) and (b), ranges from left to right as (− 4.91, 1.67, 6.50), (0, 0, 5.38), (− 5.61, − 1.90, 7.42); (d) Gravitational force *G* and the vertical component of the vector force *F*_*z*_.

The calculation process of lateral and axial component of horizontal force is similar to the principle of calculating the sum of vertical force components mentioned above.

#### Predicted muscle activity of musculoskeletal modeling

**Figure 3:**
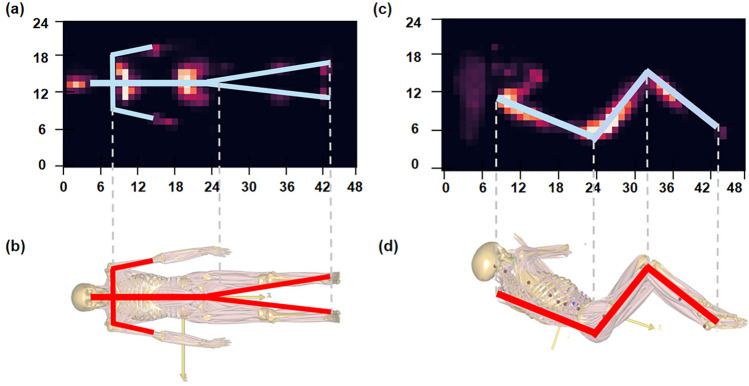
Body regions identification. The numbers in the figure represent the coordinate positions of the 24*48 sensors on the pressure mat. (**a**) 2D pressure distribution heat map in supine position, (**b**) Supine sleeping model, (**c**) 2D pressure distribution heat map in lateral position, (**d**) Lateral sleeping model.

The sleep position models were established in the AnyBody Modeling System (AnyBody Technology, Denmark). The AnyBody Managed Model Repository (AMMR 1.6.2) was adjusted based on height and weight allowed for personalization of the model^48^. The model can be scaled to match the height and weight of each participant. Referring to the supine and lateral sleeping positions of the subjects, adjustments were made to the position of the pelvis relative to the global coordinate system, as well as the flexion, abduction, rotation, and other parameters of the humerus, thighs, neck, and other body parts^49,50^. By referencing the heat map, experimental photos, and the AnyBody scale model, the corresponding input force node in AnyBody was identified for the resultant moment vector (Fig. 3). Point cloud registrations in each region were transformed into their respective moment vectors, considering both magnitude and position. Integration of this data with a 2D heat map allowed us to pinpoint the input force node in the AnyBody model. Consequently, the vector force input for both supine and lateral sleeping position AnyBody models was determined.

Based on a comparative analysis of the heat map, model, and experimental data, preliminary coordinate ranges for regions such as the head, chest, waist, hips, and legs were determined. The coordinates and vector forces corresponding to each sensor within these regions were obtained. Using the principle of resultant moments, the resultant force and resultant moment vectors for each region were calculated.

As shown in Fig. 3a and c, the lower-left corner of the pressure mat is assumed to be the origin of the coordinate system, with $\overrightarrow{r}$ representing the position vector of each sensor. For a selected area with the initial coordinate of the selected area is $\left( {x_{b} ,y_{b} } \right)$, the termination coordinate is $\left( {x_{e} ,y_{e} } \right)$, the coordinate corresponding to each point in the sensor is $\left( {x_{i} ,y_{i} } \right)$, and the vector force $\vec{F}_{si}$ of each sensor is $\left( {f_{{x_{i} }} ,f_{{y_{i} }} ,f_{{z_{i} }} } \right)$, the force couple at each sensor is calculated as follows:

7 $$\vec{M}_{i} = \vec{r} \times \mathop{F}\limits^{\rightharpoonup} \left( {b \le i \le e} \right)$$

The synergistic forces within the region are given by:

8 $$\vec{F}_{A} = \sum\limits_{i = b}^{e} {\vec{F}_{si} }$$

The resultant couple within the selected area is computed as:

9 $$\vec{M}_{A} = \vec{r}_{A} \times \vec{F}_{A} = \vec{r}_{A} \cdot \vec{F}_{A} \cdot \varepsilon_{ijk} = \sum\limits_{i = b}^{e} {\vec{M}_{i} }$$

where $\varepsilon_{ijk}$ is the permutation symbol, which represents the Eddington tensor:

10 $$\varepsilon_{ijk} = \vec{e}_{i} \cdot \left( {\vec{e}_{j} \times \vec{e}_{k} } \right)$$

Given the resultant force and resultant couple, the coordinates corresponding to the resultant force $\overrightarrow{{r}_{A}}$ can be determined:

11 $$\vec{r}_{A} = \left( {\vec{F}_{A} \cdot \varepsilon_{ijk} } \right)^{ - 1} \vec{M}_{A}$$

Thus, the resultant force and resultant moment vectors for the region are obtained.

Then, the resultant force and resultant moment vectors were input in the model as boundary condition. Inverse dynamics methods and third-order polynomial muscle supplementation criteria were used to evaluate muscle and joint forces. The predicted muscle activity in the model is defined as muscle force divided by the maximum muscle force of that specific muscle at that specific moment^28,49^.

### Data processing

The raw sEMG signals underwent filtering using a fourth-order Butterworth bandpass filter with a passband frequency range of 20-500 Hz and a notch filter at 50 Hz by the Matlab program (Matlab 2020b, Natick, Massachusetts, USA). To derive the sEMG envelope curve, a moving average root-mean-square (RMS) computation was performed on the sEMG signal, employing a window duration of 66.7 ms. To standardize the characteristic values, the peak RMS value within the MVC envelope was identified. Subsequently, the RMS during lateral and supine sleeping positions was calculated and normalized to the MVC value, thus expressing the data as a percentage of MVC (%MVC).

### Statistical analyses

Statistical analyses were conducted using SPSS Version 20.0 for Windows (SPSS Inc., Chicago, IL, USA). A paired-sample t-test was employed to ascertain whether there was a statistically significant difference between the subject's weight and the summed components of the vector force perpendicular to the ground, as determined in this experiment. The significance level was established at α = 0.05. Furthermore, to validate the efficacy of the proposed vector force input of sleeping position models, the Spearman correlation coefficient was computed between the measured muscle activity (%MVC) and the predicted muscle activity values simulated using the AnyBody sleeping models.

## Results

The vector forces exerted at the human-mattress interface computed by the mentioned method for five subjects in both supine and lateral sleeping postures are presented in Table 2. The Shapiro–Wilk test confirmed that the data for body weight, total pressure, and vertical component of vector force adhere to the normal distribution, with significance levels of 0.120, 0.097, and 0.214, respectively. The results of the independent sample t-test revealed that, across the 10 trials, there was a significant difference between the measured total force and body weight (p < 0.001). However, no significant difference was observed between the vertical force component and body weight (p > 0.05). For both supine and lateral sleeping trials, the total pressure values measured by pressure mats are 130.63% ± 6.12% (mean ± SD) times the body weight. The mean and standard deviation of the ratio of the vertical component of vector force to body weight is 98.67% ± 7.21%.

**Table 2:** Comparison of the body weight and vector force components of each subject.

Sleeping position	Subject number	Body weight (N)	Total pressure (N)	Total pressure/body weight (%)	Vertical component of vector force (N)	Vertical component force/body weight (%)	Lateral component of horizontal force(N)	Lateral component force/body weight (%)	Axial component of horizontal force(N)	Axial component force/body weight (%)
Supine	1	343.00	460.66	134.30	349.06	101.77	8.76	2.55	− 5.72	− 1.67
	2	450.80	596.15	132.24	428.79	95.12	1.66	0.37	4.46	0.99
	3	617.40	814.46	131.92	619.64	100.36	12.78	2.07	− 0.75	− 0.12
	4	676.20	843.18	124.69	655.41	96.93	− 8.89	− 1.31	8.55	1.26
	5	735.00	892.29	121.40	714.88	97.26	2.08	0.28	12.56	1.71
Lateral	1	343.00	445.74	129.95	333.68	97.28	1.32	0.38	− 13.15	− 3.83
	2	450.80	576.34	127.85	483.52	107.26	3.87	0.86	− 11.17	− 2.48
	3	617.40	872.18	141.27	685.99	111.11	− 25.50	− 4.13	− 10.84	− 1.76
	4	676.20	837.21	123.81	655.41	96.93	− 8.89	− 1.31	8.55	1.26
	5	735.00	1020.84	138.89	607.53	82.66	− 5.43	− 0.74	− 49.22	− 6.70

Regarding shear force, in the supine position, the absolute minimum value of the ratio between lateral component force and body weight was 0.28%, with a maximum value of 2.55%. The absolute minimum and maximum ratios of Axial component force to body weight were 0.12% and 1.71%, respectively. In the lateral position, the absolute minimum and maximum ratios of Lateral component force to body weight were 0.38% and 4.13%, respectively. Similarly, for the Axial component force, the absolute minimum and maximum ratios to body weight were 1.76% and 6.70%, respectively. For both supine and lateral sleeping trials of five subjects, the mean absolute value of lateral component force /body weight is 1.40% ± 1.15%, and the mean absolute value of axial component force/body weight is 2.18% ± 1.77%.

**Figure 4:**
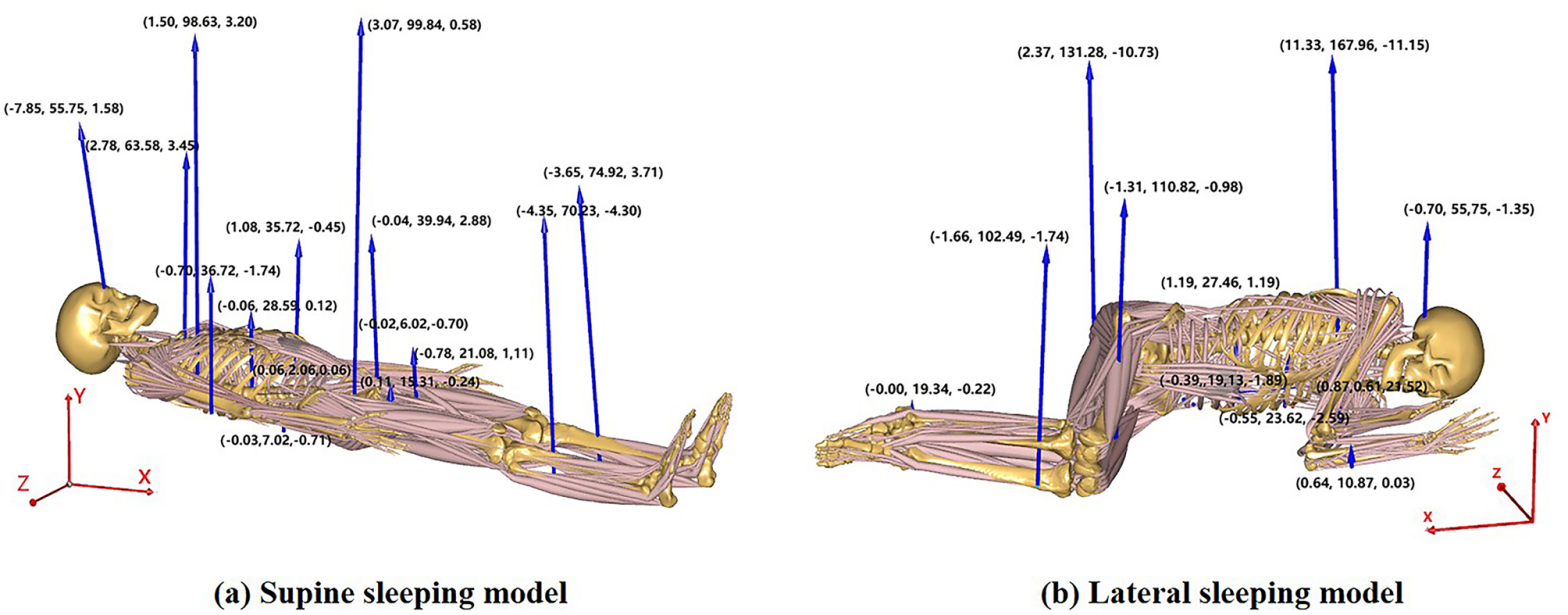
Vector force input (in Newtons) for the simulation of the AnyBody sleeping models (data of subject 4 for an example).

The vector forces calculated for each subject in both supine and lateral positions were individually inputted into their respective models (Fig. 4 illustrated the calculated vector forces of subject 4). Simulations were conducted to obtain the muscle activity of body muscles^28^.

**Figure 5:**
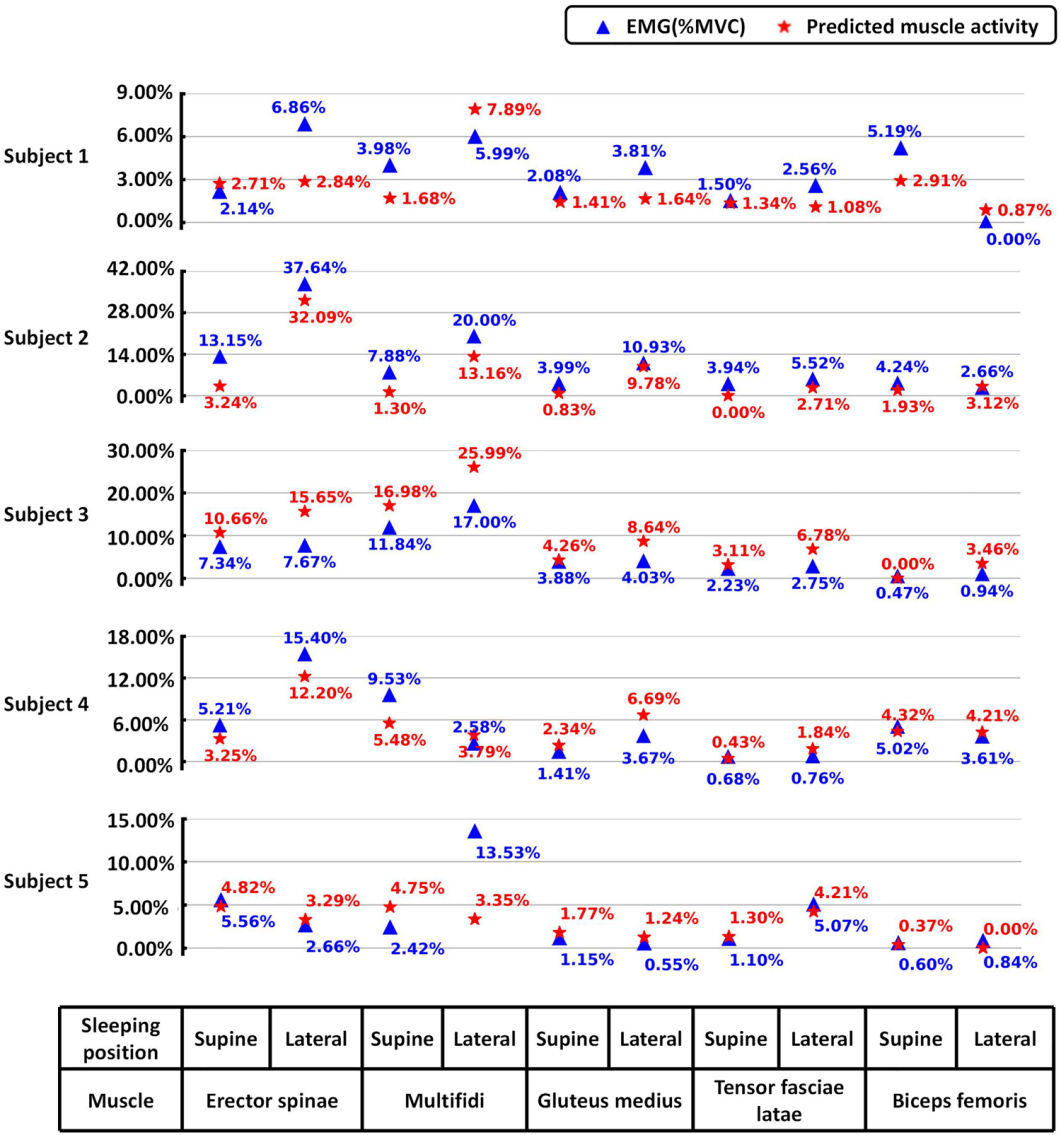
Measured muscle activity (%MVC) and predicted muscle activity values of five subjects.

Both correlation analysis and line plots indicated a significant similarity between the simulated predictions and the muscle EMG measurements. The Spearman correlation coefficient presented the relationship between the subject's measured muscle activity (%MVC) and the muscle activity values predicted by the AnyBody simulation results, which helped to confirm the effectiveness of the model. The secondary correlation analysis of the supine position (Table 3) revealed a significant correlation at the 0.01 level between %MVC and predicted muscle activity values for all 5 subjects. Figure 5 illustrated the %MVC and predicted muscle activity values for various muscles in five subjects under supine and lateral lying positions. The %MVC values closely matched the predicted EMG values, especially in the muscles of the lower body (gluteus medius, tensor fasciae latae and biceps femoris). For all subjects, the highest %MVC and model-predicted values appeared in the upper body. Specifically, Subjects 1, 3, and 5 showed peak values in multifidi, while Subject 2 and Subject 4 exhibited peak values in erector spinae.

**Table 3:** Spearman correlation coefficient and significance (double-tailed) between %MVC and predicted muscle activity values.

		Predicted muscle activity
%MVC	Subject 1	0.815 (0.004)
	Subject 2	0.770 (0.009)
	Subject 3	0.976 (< 0.001)
	Subject 4	0.821 (0.004)
	Subject 5	0.830 (0.003)

## Discussion

The current study proposed a method for calculation of vector forces between human-mattress interface. By utilizing image registration techniques on pressure distribution heat map and scanning image of vac-mat surface contour, the vector forces can be calculated. The validation was quantified in two ways.

Research into the relationship between force and sleep comfort commonly employs systems, such as pressure mats, to measure body pressure in terms of scalar force^8,51,52^. As demonstrated in Table 2, the aggregate of pressure values recorded at various locations on the pressure pad reveals that the total pressure exerted is approximately 1.3 times the subject's body weight. This pressure increase is primarily attributed to the significant deformation of the mattress surface induced by the human body, a factor that cannot be overlooked. In the present study, the pressure mat was utilized to assess the pressure distributed on the indented surface, while a vacuum positioning mattress facilitated precise measurements of surface indentations when the subject was positioned supine or laterally. By amalgamating data from these two measurements, the vector force acting on the human body was determined. Theodorakos^41^ posits that, in the context of seat optimization, maintaining mechanical balance necessitates a difference between total vertical force and subject weight of less than 5% of body weight. Aligning with this notion, the estimated vertical component of the vector force at the human-mattress interface in our study was found to be 98.6% ± 7.5% of body weight.

Regarding shear force, the present methodology yielded average ratios of 1.32% and 1.15% for the horizontal force components in the lateral and axial directions, respectively, during supine lying. These results are comparable but slightly inferior to those obtained by Beurier et al.^44^ in their assessment of measured contact forces using a Multi-Adjustable Experimental Seat. In their study, the sum of the horizontal forces for the subjects ranged between 0.07% and 1.07% of body weight, due to a more intricate sensor design in the seat. For the five subjects in our study, the average ratios of lateral and axial components of horizontal force to body weight during side lying were 1.49% and 3.21%, respectively, showing higher values than the system developed by Cho et al.^53^. Their shear pad composed of 24 sensors and could directly measure shear force alone at human–machine interfaces, with measuring shear force even at correlated low values (0.3N of shear force). However, the testing pad has a small area and cannot be used for measuring the shear force of the entire mattress. The occurrence of relatively large shear forces in this study may be due to the asymmetrical nature of side lying, which can lead to higher pressure between the body and the mattress, potentially resulting in greater static friction.

The accuracy of the predicted outcomes obtained from the sleeping posture model can be validated through participant involvement. To ascertain the precision and feasibility of the muscle model, sEMG data were collected from the participants and compared with the muscle activity values estimated by the model. In the supine posture, the Spearman correlation coefficients (Table 3) between the %MVC and predicted muscle activity values for all five participants demonstrated a strong positive correlation, above 0.7. This suggests that the model's muscle predictions are reliable, indirectly indicating the feasibility of the model's input values, namely, the vector forces calculated using the methodology of this study.

Furthermore, based on the results of simulation predictions, it was observed that for the five subjects, paraspinal muscles exhibit higher activity during sleep compared to thigh muscles (Fig. 5). Yu-chi et al.^20^ collected electromyography data from the trapezius, lumbar paraspinal, biceps femoris, and gastrocnemius muscles of subjects in a supine position. They estimated the %MVC for each muscle, providing valuable insights into muscle activity during sleep. Similarly, Jacobson et al.^54^ recorded electrical activity from trunk muscles (pectoralis, latissimus dorsi, serratus anterior, rectus abdominis, and external oblique) and leg muscles (gluteus medius, adductor longus, sartorius, rectus femoris, biceps femoris, tibialis anterior, and gastrocnemius) during sleep. Although different methods were used to assess muscle activity, they consistently indicate that paraspinal muscle activity is greater than thigh muscle activity during sleep. Our simulation predictions indicate that, except for Subject 1(muscle activity of erector spinae is 2.71%; biceps femoris is 2.91%), the maximum muscle activity during supine lying occurs in the erector spinae or multifidus muscles of the lower back, with activity values reaching up to 16.98%. In the left lateral lying position, the highest muscle activity is observed in the upper body muscles, with a minimum value of 7.89% and a maximum of 32.09% aside from subject 5, This suggests that during lateral lying, the contralateral lower back muscles are in a tense state, resulting in increased pressure on the lower back compared to supine lying. Moreover, the choice of lateral lying direction differentially affects the muscle load on both sides of the lower back.

The analysis results of sEMG eigenvalue experiments further explain the above findings. As shown in Fig. 5, during lateral lying, the muscle activity in the lower body muscles (gluteus medius, tensor fasciae latae and biceps femoris) is generally lower than that in the paraspinal muscles (ereator spinae and multifidi). This is because the vacuum-positioned mattress used in this study is designed to be soft, providing better support for the muscles of the thighs and promoting relaxation. However, maintaining a posture against gravity involves both paraspinal and leg muscles^55^, relying primarily on the waist muscles to maintain the lateral lying position. It is worth noting that softer beds often lead to more significant hip sinking, which can also cause the lumbar spine to sink. When lying on one side, the limited contact area between the body and the mattress leads to further sinking of the hips. These factors may contribute to less effective relaxation of the upper body muscles when sleeping on a soft mattress, explaining why complaints about sleep-related mechanical fatigue mainly occur in the upper body. Therefore, mattress designers should consider the mechanical relationship between the human body and the mattress in different sleeping positions when designing the softness and stiffness of the mattress.

### Limitations

Although this study included both male and female participants to validate the calculation method of vector force at the human-mattress interface, the BMI values of the participants were all below 25, and the feasibility of this method for overweight individuals was not verified. Therefore, future research should aim to supplement this aspect of validation because people with different body fat distributions may have varying sleep comfort needs^56^. Additionally, while this study scanned the contour of the vacuum mattress after the participant lay down, which could be used to study the back shape of individuals in a supine position, it did not capture the back shape of individuals in a natural standing position for comparison with the scanned surface indentations. We believe that future analyses based on this method, comparing the different alignments of the chest and lumbar spine between sleeping and standing positions, would be beneficial and could further enhance the accuracy of vector force calculations.

## Conclusion

This study introduces a method for calculating vector forces at the human-mattress interface using vacuum positioning mattresses and image registration techniques. The effectiveness of this method was validated through experiments with five subjects. This method delivers precise vector force inputs for musculoskeletal models of sleeping postures, thereby aiding in the mechanical simulation of musculoskeletal systems and presenting a non-invasive approach for analyzing muscle force and joint torque. This holds significant value in assessing the muscle relaxation effects of various mattresses and guiding the design of optimal ones.

## Data Availability

The datasets used and/or analysed during the current study are available from the corresponding author on reasonable request.
